# Supplementary material for: Rational social distancing policy during epidemics with limited healthcare capacity
Source: PLoS Comput Biol. 2023 Oct 16;19(10):e1011533. doi: 10.1371/journal.pcbi.1011533 (PMC10602387; doi:10.1371/journal.pcbi.1011533)
Supplement: S1 Text — (PDF) [file pcbi.1011533.s001.pdf]

Supporting Information:
Rational social distancing policy during epidemics with limited healthcare capacity

Simon K. Schnyder[1*], John J. Molina[2], Ryoichi Yamamoto[2], Matthew S. Turner[3,4]

1 Institute of Industrial Science, The University of Tokyo, Tokyo, Japan
2 Department of Chemical Engineering, Kyoto University, Kyoto, Japan
3 Department of Physics, University of Warwick, Coventry, UK
4 Institute for Global Pandemic Planning, University of Warwick, Coventry, UK

* skschnyder@gmail.com

Contents

A Calculus of variations **1**
A.1 Euler-Lagrange equations 1
A.1.1 Payoff 2
A.1.2 Fixed end points 2
A.1.3 Variable end point 3
A.1.4 Variable end time 3
A.1.5 Constraints 3
A.2 Hamilton's equations 4

B Optimal control theory **5**
B.1 Hamiltonian formulation 6
B.2 Pontryagin's principle 8
B.3 Bounded controls – constrained optimisation 9

C Forward-backward sweep method **10**

D Utility salvage term for vaccination at t_f **12**

A Calculus of variations

A.1 Euler-Lagrange equations

Consider a functional $S[q]$, i.e., a function S of a (path) function $q(t)$. We are interested in linear functionals, expressed as integrals of a function L over a given time interval (t_1, t_2). We will assume that L can depend on time t, the value of the path $q(t)$, and the (time) derivative of the path $\dot{q}(t) \equiv \mathrm{d}q(t)/\,\mathrm{d}t$, such that

$$S[q](t_1, t_2) = \int_{t_1}^{t_2} L(t, q(t), \dot{q}(t))\,\mathrm{d}t = \int_{t_1}^{t_2} L(t, q, \dot{q})\,\mathrm{d}t = \int_{t_1}^{t_2} L\,\mathrm{d}t \tag{S1}$$

To find the minimum/maximum with respect to the path, we must extremise this functional. This is done by requiring that the variations in S, with respect to the path,

vanish to first-order. Let the variation in the path be given by $q \to q + h$, the corresponding variation in S is [1,2]

$$\big(S[q+h] - S[q]\big)(t_1, t_2) = \int_{t_1}^{t_2} \Big[L(t, q+h, \dot{q}+\dot{h}) - L(t, q, \dot{q})\Big] \tag{S2}$$

$$= \int_{t_1}^{t_2} \Big[(\partial_q L)\, h + (\partial_{\dot{q}} L)\, \dot{h}\Big]\, \mathrm{d}t + \mathcal{O}(h^2) \tag{S3}$$

Integrating the second term in the integral by parts, the variation in S is then

$$\delta_h S[q] = \int_{t_1}^{t_2} \left(\partial_q L - \frac{\mathrm{d}}{\mathrm{d}t} \partial_{\dot{q}} L\right) h\, \mathrm{d}t + (\partial_{\dot{q}} L)\, h\Big|_{t_1}^{t_2} \tag{S4}$$

Optimal paths are those for which the functional is extremised, i.e.,

$$\delta_h S[q](t_1, t_2) = 0 \tag{S5}$$

Since h is arbitrary, except possibly at the end-points, we will require that both terms vanish. In particular, this means that the integrand of the first term must be exactly zero, which will yield the Euler-Lagrange (EL) equations. The second (boundary) term, provides the so-called *transversality* conditions, which may or may not be necessary, depending on the boundary conditions that are imposed, i.e., whether $h(t_1)$ and $h(t_2)$ vanish.

We note that within a Classical mechanics setting [2,3], S would be the "action" integral, L the Lagrangian of the system, and $q(t)$ the coordinate path function ($\dot{q}(t)$ the velocity along the path). The "principle of least (stationary)-action" states that the physically realisable paths q are those that extremise the action, i.e., $\delta_h S[q](t_1, t_2) = 0$.

A.1.1 Payoff

Within an Optimal Control framework [4,5], it is common to associate a cost ϕ to the end-state of the system. The (action) functional to be extremised is then $S_\phi[q](t_1, t_2) = S[q](t_1, t_2) + \phi(t_2, q(t_2))$. However, this can be also be expressed in the standard form of Eq. (S1), since

$$S_\phi[q](t_1, t_2) = \int_{t_1}^{t_2} \big(L(t, q, \dot{q}) + \phi(t, q)\, \delta(t - t_2)\big)\, \mathrm{d}t \tag{S6}$$

The variation of S_ϕ is again given by Eq. (S4), and can be written as

$$\delta_h S_\phi[q](t_1, t_2) = \int_{t_1}^{t_2} \left(\partial_q L - \frac{\mathrm{d}}{\mathrm{d}t} \partial_{\dot{q}} L\right) h\, \mathrm{d}t + (\partial_{\dot{q}} L)\, h\Big|_{t_1}^{t_2} + (\partial_q \phi)\, h\Big|_{t_2} \tag{S7}$$

$$= \delta_h S[q](t_1, t_2) + (\partial_q \phi)\, h\Big|_{t_2} \tag{S8}$$

where we have picked up an additional boundary term at $t = t_2$.

A.1.2 Fixed end points

Let us first consider the simplest variational problem, which is that of fixed end-points. If the path is constrained to be such that $q(t_1) = q_1$ and $q(t_2) = q_2$, then the variations in the path must vanish at the end-points, $h(t_1) = h(t_2) = 0$, so that the varied path $q + h$ also satisfies the constraints. In this case, the boundary terms vanish exactly (regardless of the payoff function), and the stationary condition for the action reduces to the set of Euler-Lagrange equations [1]

$$\frac{\mathrm{d}}{\mathrm{d}t} \partial_{\dot{q}} L - \partial_q L = 0 \tag{S9}$$

A.1.3 Variable end point

Now consider the case where one of the end-points is free. For the purposes of this work, we focus on problems where the initial state is fixed $q(t_1) = q_1$, but the end-state $q(t_2)$ is free. Thus, the only constraint on the variation is that $h(t_1) = 0$, with $h(t_2)$ arbitrary. The stationary condition now requires the EL equations to be satisfied, together with an additional boundary condition at the end-state [1]

$$\frac{\mathrm{d}}{\mathrm{d}t}\partial_{\dot{q}}L - \partial_q L = 0 \tag{S10}$$

$$\left.(\partial_{\dot{q}}L + \partial_q \phi)\right|_{t_2} = 0 \tag{S11}$$

Here, since the end-state is not fixed, the payoff term $\phi(t_2, q(t_2))$ does play a role.

A.1.4 Variable end time

For completeness, even though it is not required in our present study, we can also consider variations in the end-time. The variation of the S functional must now account for variations in both the path q and the end-time t_2. To first-order, this is simply [1]

$$\delta S[q](t_1, t_2) = \delta_h S[q](t_1, t_2) + \delta_{t_2} S[q](t_1, t_2) \tag{S12}$$

where $\delta_h S[q]$ is given by Eq.(S4) and the time-variation is

$$\delta_{t_2} S[q](t_1, t_2) = \frac{\mathrm{d}}{\mathrm{d}t_2}\int_{t_1}^{t_2} L\,\mathrm{d}t = L(t_2, q(t_2), \dot{q}(t_2)) \tag{S13}$$

such that the stationary condition becomes

$$\frac{\mathrm{d}}{\mathrm{d}t}\partial_{\dot{q}}L - \partial_q L = 0 \tag{S14}$$

$$\left.(\partial_{\dot{q}}L + \partial_q \phi + L)\right|_{t_2} = 0 \tag{S15}$$

A.1.5 Constraints

Under certain conditions, the above formulation can be extended to constrained optimisation/extremisation problems, i.e., extremising the functional $S[q]$ subject to a constraint on the path function of the form

$$\psi(t, q, \dot{q}) = 0. \tag{S16}$$

While a general solution is not known, if the constraint is integrable or holonomic, that is, if it is a coordinate constraint (no-velocity dependence) or the time-derivative of a coordinate constraint, then the constrained optimisation can be expressed as an unconstrained optimisation. This is done by introducing Lagrange multipliers λ to define an "augmented" Lagrangian L' [2,4]

$$L' = L + \lambda\psi. \tag{S17}$$

Here, the Lagrange multipliers λ enter L' as additional "coordinate" degrees of freedom (on the same level as q). The augmented Lagrangian $L' = L'(t, q', \dot{q}')$ is a function of the augmented coordinates q' and velocities $\dot{q}'$, where $q' = (q, \lambda)$.

The solution to the (original) constrained extremisation problem is then given by the solution to the unconstrained extremisation defined by the augmented Lagrangian L'

$$\delta_{h'} S'[q](t_1,t_2) = \delta_{h'} \left[\int_{t_1}^{t_2} L' \, \mathrm{d}t + \phi(t_2, q(t_2)) \right] = 0 \tag{S18}$$

The EL equations for the q degrees of freedom are given by Eq. S10

$$\frac{\mathrm{d}}{\mathrm{d}t} \partial_{\dot{q}} L' - \partial_q L' = 0 \tag{S19}$$

whereas the EL equations for the augmented degrees of freedom (the Lagrange multipliers λ) simply recover the constraint, as expected,

$$\frac{\mathrm{d}}{\mathrm{d}t} \partial_{\dot{\lambda}} L' - \partial_\lambda L' = \psi = 0 \tag{S20}$$

Finally, since the augmented Lagrangian L' has no $\dot{\lambda}$ dependence ($\partial_{\dot{\lambda}} L' = 0$), and the payoff function does not explicitly depend on λ ($\partial_\lambda \phi = 0$), we only obtain transversality conditions from the original q degrees of freedom. Assuming a variable end point, but a fixed end time, Eq. S11 becomes

$$\left(\partial_{\dot{q}} L' + \partial_q \phi \right)\big|_{t_2} = 0 \tag{S21}$$

A.2 Hamilton's equations

The EL equations, obtained by extremising the action S, i.e. the integral of the Lagrangian, specify the dynamics of a dynamical system in terms of the coordinates q and velocities $\dot{q}$. An alternative to this Lagrangian formulation is given by the Hamiltonian formulation, which specifies the dynamics in terms of the coordinates q and momenta p, where the momentum on a path is defined as [2]

$$p(t) = \partial_{\dot{q}} L(t, q, \dot{q}) \tag{S22}$$

such that the EL equations can be written as

$$\frac{\mathrm{d}}{\mathrm{d}t} p(t) = \partial_q L(t, q(t), \dot{q}(t)) \tag{S23}$$

To obtain Hamilton's equations, we eliminate $\dot{q}$ in terms of p. Let $\mathcal{V}$ be the function that provides the velocities in terms of time, coordinates, and momenta, i.e., $\mathcal{V}(t, q(t), p(t)) = \dot{q}(t)$. The Hamiltonian H of the system, corresponding to the Lagrangian L, is given by the following Legendre transform [2, 3],

$$H(t, q, p) = p(t)\mathcal{V}(t, q, p) - L(t, q, \mathcal{V}(t, q, p)) \tag{S24}$$

and Hamilton's equations, describing the time-evolution of the system, now in terms of q and p, are [2, 3]

$$\dot{q}(t) = \partial_p H \tag{S25}$$

$$\dot{p}(t) = -\partial_q H \tag{S26}$$

The transversality condition, Eq.(S11), becomes

$$\left(p + \partial_q \phi \right)\big|_{t_2} = 0 \tag{S27}$$

We note that both the Lagrangian and Hamiltonian formulations are equivalent, in the sense that they will reproduce identical trajectories given identical starting conditions. Furthermore, as we will see below, for the type of optimal control problems we are interested in, they are not only equivalent, but they result in exactly the same set of equations.

B Optimal control theory

The goal of this section is to state the optimal control problem relevant for this work, which can be considered as a specific case of the variational problem detailed in section Calculus of variations above, see also [5, 6].

Consider a system whose state at time t is given by $x(t) = (x^1(t), ..., x^n(t))$ (i.e., x are the coordinates), and whose dynamics is constrained to satisfy the following ordinary differential equation (with fixed initial condition)

$$\dot{x}(t) = F(t, x(t), a(t)), \qquad x(0) = x_0 \tag{S28}$$

$$\dot{x}^i(t) = F^i(t, x(t), a(t)) \tag{S29}$$

where $a(t) = (a^1(t), ..., a^m(t))$ is a control function that the system can independently "control", thus providing additional degrees of freedom. Let U be a scalar functional measuring the total utility/cost associated with a given trajectory (i.e., the action),

$$U[x](t_0, t_f) = \int_{t_0}^{t_f} C(t, x(t), a(t))\, \mathrm{d}t + \phi(t_f, x(t_f)) \tag{S30}$$

with $C(t, x(t), a(t))$ the running cost per time (i.e., the Lagrangian) and $\phi(t_f, x(t_f))$ the cost at the end time (i.e., the payoff or salvage term).

The dynamical constraint $\dot{x} = F$ is an example of an integrable/holonomic constraint, since it can be expressed as a coordinate constraint

$$x(t) = x_0 + \int_{t_0}^{t} F(t, x(t), a(t))\, \mathrm{d}t \tag{S31}$$

Thus, this constrained optimisation problem can be reformulated as an unconstrained optimisation over for an augmented Lagrangian $L(t, q, \dot{q})$, with generalised coordinates $q = (x, a, \lambda)$. Following Eq. (S17), with $\psi(t, x, \dot{x}) = F(t, x) - \dot{x}$, we have

$$L(t, q, \dot{q}) = C(t, x, a) + \lambda_i \left(F^i(t, x, a) - \dot{x}^i\right) \tag{S32}$$

where $\lambda_i(t) = (\lambda_1(t), \lambda_2(t), \cdots, \lambda_n(t))$, i.e., there is one Lagrange multiplier for each constrained degree of freedom, and the Einstein summation convention is used $(\sum a_i b^i \equiv a_i b^i)$. Within the optimal control literature, these Lagrange multipliers are referred to as *adjoint* or *co-state* variables (we use these terms interchangeably) [4]. The Euler-Lagrange equations corresponding to this optimisation problem are given by Eqs. (S19-S21)

$$\frac{\mathrm{d}}{\mathrm{d}t}\frac{\partial L}{\partial \dot{q}^\alpha} = \frac{\partial L}{\partial q^\alpha} \qquad \alpha = 1, \ldots, 2n + m \tag{S33}$$

$$\left.\left(\partial_{\dot{x}^i} L + \partial_{x^i}\phi\right)\right|_{t_f} = 0 \qquad i = 1, \ldots, n \tag{S34}$$

where $q^i = x^i$, $q^{n+j} = a^j$, and $q^{n+m+i} = \lambda_i$ $(1 \leq i \leq n,\ 1 \leq j \leq m)$. To derive these equations we have considered variations in the "path" q, which includes the state x, the

control a, and the co-state λ. We note that we are considering the case where only the initial state is fixed $x(t_0) = x_0$, there is no such constraint on the initial value of a or λ. For this reason, we only obtain transversality conditions for the x degrees of freedom.

Given the precise form of the Lagrangian we use, where the velocities only enter through the constraint term, the EL equations for the state (x) degrees of freedom, $1 \leq \alpha \leq n$, result in a set of ordinary differential equations for the co-state (λ) dynamics (together with end-time boundary conditions)

$$\frac{\mathrm{d}}{\mathrm{d}t}\lambda_i = \dot{\lambda}_i = -\partial_{x^i} L \tag{S35}$$

$$\lambda_i(t_f) = \partial_{x^i}\phi(t_f, x(t_f)) \tag{S36}$$

Furthermore, since the Lagrangian has no dependence on the control velocities, the corresponding EL equations reduce to an optimality condition for the control,

$$\frac{\partial L}{\partial a^j} = 0. \tag{S37}$$

Finally, the EL equations for co-state, $n + m \leq \alpha \leq 2n + m$, simply reproduce the constraint dynamics

$$\dot{x}(t) = F \tag{S38}$$

In summary, the solution to any optimal control problem of the form

$$a_{\text{opt}}(t) = \arg\min_a \left[\int_{t_0}^{t_f} C(t, x(t), a(t)) + \phi(t_f, x(t_f))\right] \tag{S39}$$

$$\text{such that} \quad \dot{x}(t) = F(t, x(t), a(t)) \tag{S40}$$

$$x(t_0) = x_0 \tag{S41}$$

can be found by integrating the following set of ordinary differential equations

$$\dot{x}(t) = F(t, x, a_{\text{opt}}) \tag{S42}$$

$$\dot{\lambda}(t) = -\partial_{x^i} L(t, x, a_{\text{opt}}) \tag{S43}$$

$$L(t, x, a, e) = C(t, x, a) + \lambda_i(F^i(t, x, a) - \dot{x}^i) \tag{S44}$$

where the optimal control is defined such that

$$\left(\partial_a L\right)(t, x, a_{\text{opt}}) = 0 \tag{S45}$$

under mixed boundary conditions for the initial state $x(t_0)$ and the final co-state $\lambda(t_f)$

$$x(t_0) = x_0 \tag{S46}$$

$$\lambda(t_f) = \partial_{x^i}\phi(t_f, x(t_f)) \tag{S47}$$

B.1 Hamiltonian formulation

To formulate the dynamics within the Hamiltonian formalism, we compute the appropriate Hamiltonian, with x, a the active coordinates in the Legendre transform (λ

being passive) [2]

$$
\begin{aligned}
H(t,q,p) &= p_i \mathcal{V}^i(t,q,p) - L(t,q,\mathcal{V}(t,q,p)), \qquad 1 \le i \le n && \text{(S48)}\\
&= p_i \mathcal{V}^i(t,q,p) - C(t,x,a) - \lambda_i \left(F^i(t,x,a) - \mathcal{V}^i(t,q,p)\right) && \text{(S49)}\\
&= \mathcal{V}^i(t,q,p)\,(p_i + \lambda_i) - C(t,x,a) - \lambda_i F^i(t,x,a) && \text{(S50)}\\
&= -\left(C(t,x,a) + \lambda_i F^i(t,x,a)\right) && \text{(S51)}\\
&= -C(t,x,a) + p_i F^i(t,x,a) && \text{(S52)}
\end{aligned}
$$

where we have used the fact that the Lagrange multipliers λ are equal (up to a sign) to the momenta conjugate to the state coordinates x, and the momenta conjugate to a are exactly zero, since

$$
\begin{aligned}
p_{x^i} &= \partial_{\dot{x}^i} L = -\lambda_i && \text{(S53)}\\
p_{a^j} &= \partial_{\dot{a}^j} L = 0 && \text{(S54)}
\end{aligned}
$$

For simplicity, we will avoid introducing the additional momentum variables p, since they are equivalent to the Lagrange multipliers λ. Furthermore, following the optimal control literature, we will change the sign of H, such that it is minimised [4]. Thus, we define the *control* Hamiltonian as

$$
H(t,q,\lambda) = C(t,x,a) + \lambda_i F^i(t,x,a) \tag{S55}
$$

i.e., it is equal to minus the physics Hamiltonian. For what follows, and throughout the main text, any reference to the Hamiltonian will refer to this control Hamiltonian.

In this way, the (control) Hamiltonian only depends on the generalised coordinates (x,a) and the Lagrange multipliers λ. The latter no longer play the role of coordinates λ, but instead assume the role of momenta. We note that this is only true because the cost function C has no explicit dependence on the velocities. The velocities only enter the augmented Lagrangian (linearly) in the form of the constraint. Furthermore, this Lagrangian only depends on the velocities associated to the state x, and not those of the control or Lagrange multipliers.

Hamilton's equations for the state degrees of freedom are then

$$
\begin{aligned}
\dot{x}^i &\equiv \partial_{\lambda_i} H = F^i && \text{(S56)}\\
\dot{\lambda}_i &\equiv -\partial_{x^i} H = \partial_{x^i}\left(C + \lambda_i F^i\right) && \text{(S57)}
\end{aligned}
$$

where the first equation recovers the constraint dynamics and the second equation is exactly the EL equation for the co-state variables. The remaining equations for the control degrees of freedom (i.e. the optimality conditions) are obtained from $p_a \equiv \partial_{\dot{a}} H = 0$

$$
\dot{p}_a(t) \equiv \partial_a H = \partial_a \left(C + \lambda_i F^i\right) = 0 \tag{S58}
$$

which are, as expected, exactly the optimality conditions obtained within the Lagrangian picture, since

$$
\begin{aligned}
\partial_a L &= \partial_a (C + \lambda_i F^i - \lambda_i \dot{x}^i) && \text{(S59)}\\
&= \partial_a (C + \lambda_i F^i) && \text{(S60)}
\end{aligned}
$$

To summarise, the optimal control problem of Eqs.(S39-S41) is solved by the following set of (Hamiltonian) equations

$$
\begin{aligned}
\dot{x}(t) &= F(t,x,a_{\text{opt}}) && \text{(S61)}\\
\dot{\lambda}(t) &= -\partial_x H(t,x,a,\lambda) && \text{(S62)}\\
H(t,x,a,\lambda) &= C(t,x,a) + \lambda_i F^i(t,x,a) && \text{(S63)}
\end{aligned}
$$

with the optimal control determined by the condition

$$\partial_a H(t, x, a_{\text{opt}}, \lambda) = 0 \tag{S64}$$

under the mixed set of boundary conditions

$$x(t_0) = x_0 \tag{S65}$$
$$\lambda(t_f) = \partial_x \phi(t, x(t_f)) \tag{S66}$$

B.2 Pontryagin's principle

Recall the optimisation problem we wish to solve,

$$\text{minimise} \left[\int_{t_0}^{t_f} C(t, x(t), a(t)) + \phi(t_f, x(t_f)) \right] \tag{S67}$$
$$\text{such that} \qquad \dot{x}(t) = F(t, x(t), a(t)) \tag{S68}$$
$$x(t_0) = x_0 \tag{S69}$$

Until now, our variational approach assumed no constraints on the paths (except possibly at the end-points), as well as a continuous control a. For the case in which the controls are not arbitrary, but must instead be chosen from some allowed set $a \in \mathcal{A}$, we require a more general formalism to establish the optimality conditions. These are provided by Pontryagin's Principle, which encompass the Euler-Lagrange equations [5, 7].

Pontryagin's Principle states that there exist costate variables λ such that

$$\dot{x}(t) = \partial_\lambda \mathcal{H} \tag{S70}$$
$$\dot{\lambda}(t) = -\partial_x \mathcal{H} \tag{S71}$$

with mixed boundary conditions

$$x(t_0) = x_0 \tag{S72}$$
$$\lambda(t_f) = \partial_x \phi(t_f, x(t_f)) \tag{S73}$$

where $\mathcal{H}$ is defined in terms of the Hamiltonian ($H = C + \lambda_i F^i$) as

$$\mathcal{H}(t, x, \lambda) = \min_{a \in \mathcal{A}} H(t, x, a, \lambda) = H(t, x, a^\star, \lambda) \tag{S74}$$
$$a^\star = \arg\min_{a \in \mathcal{A}} H(t, x, a, \lambda) \tag{S75}$$

with $a^\star$ the optimal control. This optimality condition can also be expressed as

$$H(t, x, a^\star, \lambda) \leq H(t, x, a, \lambda) \tag{S76}$$

We see that the Pontryagin Principle encompasses the Euler-Lagrange / Hamiltonian equations derived above, by replacing the optimality condition ($\partial_a H = 0$), with Eq. S75, which states that the optimal control $a^\star \in \mathcal{A}$ is that which minimises the Hamiltonian. This minimisation over a, which must be performed at each time, is then reduced to a standard non-linear constrained optimisation problem [8].

B.3 Bounded controls – constrained optimisation

For simplicity, since the optimality condition is expressed only in terms of a, denote $f(a) = H(t, x, a, \lambda)$. Let the constraints on a be given by a series of inequality constraints c_i, such that the optimal control is determined by

$$a^\star = \arg\min_a f(a) \qquad \text{such that}\, c_i(a) \geq 0 \tag{S77}$$

This constrained extremisation problem can again be solved by introducing additional Lagrange multipliers μ, to define the Lagrangian $\mathcal{L}(a, \mu) = f(a) - \sum_i \mu_i c_i(a)$. Under appropriate regularity conditions, or qualifications on the constraints c_i, the necessary (first-order) conditions for optimality are the so-called Karush-Kuhn-Tucker (KKT) conditions [8]

$$\partial_a \mathcal{L}(a^\star, \mu^\star) = 0 \tag{S78}$$
$$c_i(a^\star) \geq 0 \tag{S79}$$
$$\mu_i^\star \geq 0 \tag{S80}$$
$$\mu_i c_i(a^\star) = 0 \tag{S81}$$

The last equation, the so-called *complementarity* condition, states that either $c_i = 0$, in which case the constraint is active, or $\mu_i = 0$ and the constraint is inactive. Thus, in general, we need to solve for both a and μ.

In this work we are interested in bounded 1-d controls,

$$a_{\text{min}} \leq a \leq a_{\text{max}} \tag{S82}$$

such that we have two (affine) constraints

$$c_1(a) = a - a_{\text{min}} \tag{S83}$$
$$c_2(a) = -a + a_{\text{max}} \tag{S84}$$

This is an example of a linearity constraint qualification (LCQ), which guarantees that the solutions to the constrained optimisation problem satisfy the KKT conditions [8]. If the minimiser $a^\star$ lies inside the domain ($a_{\text{min}} < a < a_{\text{max}}$), such that both constraints are inactive ($\mu_1^\star = \mu_2^\star = 0$), we see that the solution to the constrained optimisation coincides with that of the unconstrained one, $\partial_a \mathcal{L}(a^\star, 0) = \partial_a f(a^\star) = 0$. Furthermore, we see that the constraints are mutually exclusive, as at most one of them can be activated ($a_{\text{min}} < a_{\text{max}}$). If c_1 is the active constraint, we have

$$c_1(a^\star) = a^\star - a_{\text{min}} = 0 \tag{S85}$$
$$a^\star = a_{\text{min}} \tag{S86}$$
$$\mu_1^\star = \partial_a f(a_{\text{min}}) \tag{S87}$$
$$\mu_2^\star = 0 \tag{S88}$$

whereas for c_2 we have

$$c_2(a^\star) = -a^\star + a_{\text{max}} = 0 \tag{S89}$$
$$a^\star = a_{\text{max}} \tag{S90}$$
$$\mu_2^\star = -\partial_a f(a_{\text{max}}) \tag{S91}$$
$$\mu_1^\star = 0 \tag{S92}$$

The (active) Lagrange multipliers are obtained from the optimality condition (first KKT condition)

$$\partial_a \mathcal{L}(a^\star, \mu^\star) = \partial_a f(a^\star, \mu^\star) - \sum_i \mu_i^\star \partial_a c_i(a^\star) = 0 \tag{S93}$$

but for this particularly simple set of inequality constraints, we have no use for their values. Note that the gradient of f is positive (negative) at the lower (upper) bounds, as expected.

Computationally, we can compute the optimal control as follows. Let $a^\dagger$ be the minimiser for the unconstrained problem,

$$a^\dagger = \arg\min_a f(a) \tag{S94}$$

the (constrained) solution $a^\star$ is

$$a^\star = \begin{cases} a_{\min} & a^\dagger \leq a_{\min} \\ a^\dagger & a_{\min} < a^\dagger < a_{\max} \\ a_{\max} & a^\dagger \geq a_{\max} \end{cases} \tag{S95}$$

$$= \min\left(\max\left(a_{\min}, a^\dagger\right), a_{\max}\right) \tag{S96}$$

C Forward-backward sweep method

We seek to numerically solve the optimal control problem

$$\text{maximise} \left[\int_{t_0}^{t_f} C(t, x(t), a(t))\,\mathrm{d}t + \phi(t_f, x(t_f))\right]$$

$$\text{subject to } \dot{x}(t) = F(t, x(t), a(t)), \text{ with } x(t_0) = x_0, \tag{S97}$$

$$a_{min} \leq a(t) \leq a_{max}, \text{ for all } t \in [t_0, t_f] \tag{S98}$$

which we can recast with the help of section Optimal control theory of the SI as a boundary value problem involving the Hamiltonian

$$H(t, x(t), a(t), v(t)) = C(t, x(t), a(t)) + v(t) \cdot F(t, x(t), a(t)) \tag{S99}$$

The boundary value problem reads, with Lagrange multipliers $v(t)$

$$\dot{x}(t) = F(t, x(t), a(t)), \text{ with } x(t_0) = x_0 \tag{S100}$$

$$\dot{v}(t) = -\partial_x H(t, x(t), v(t), a(t)), \text{ with } v(t_f) = \partial_x \phi(t_f, x(t_f)) \tag{S101}$$

The optimality condition on the unconstrained control $a^\dagger$

$$0 = \partial_{a^\dagger} H(t, x(t), v(t), a^\dagger(t)) \tag{S102}$$

can typically, and certainly in our case, be explicitly solved for $a^\dagger$. The optimal bounded control then directly follows

$$a = \min\left(\max\left(a_{\min}, a^\dagger\right), a_{\max}\right) \tag{S103}$$

For the algorithm, we can exploit the special structure of this problem: the dynamics F don't depend on the Lagrange multipliers and can therefore be solved without their knowledge. Furthermore, the boundary conditions are unmatched, with the dynamics having one at the initial time, whereas the Lagrange multipliers have one at the final time. This structure allows for constructing a special solver, the forward-backward sweep method [6]: Then, we perform the following steps,

1. **Guess a control:** We make an initial guess for the control $a(t)$ on the whole time interval, $t \in [t_0, t_f]$.

2. **Solve dynamics forward:** Given the control $a(t)$, we calculate the course of the dynamics $x(t)$ over the time interval by integrating eq. (S100) *forward* in time, starting from t_0.

3. **Solve Lagrange multipliers backward:** Given the course of the dynamics $x(t)$, the control $a(t)$, we calculate the corresponding Lagrange multipliers $v(t)$ by integrating eq. (S101) *backward* in time, starting from t_f.

4. **Calculate new control:** Rename $a(t)$ to $a_{old}(t)$. Given the course of the dynamics $x(t)$, the Lagrange multipliers $v(t)$, we can calculate a new control $a_{new}(t)$ using eq. (S103). In practice, however, it is not ideal to directly use the newly calculated values for the control, but instead calculate a convex combination of a_{new} and a_{old} to improve convergence. Since the bounds only need to be imposed on the new iteration of the control, we calculate a_{new} without applying bounds and then calculated a bounded linear combination of the old and new controls, with a mixing parameter m:

$$a = \min\left(\max\left(a_{min}, (1-m)\, a_{old} + m\, a_{new}\right), a_{max}\right) \tag{S104}$$

   We found that we had to be careful with the choice of m. A small value meant that the algorithm would take many iterations to converge, whereas a large value meant that the algorithm would never converge, as it would keep overshooting the optimal control and therefore oscillate around it. As a result we used values between $m = 0.005$ and $m = 0.0005$ for the population behaviour, and $m = 0.0005$ for the government intervention.

5. **Check for convergence:** We considered the algorithm to have converged when the control and the system's dynamics when each of the relative changes in x and a in one iteration step were smaller than a precision threshold Δp. In order to avoid issues for when the norm on the old result might turn out to be 0 by coincidence, we implemented

$$\Delta p \, \|x_{old}\|_1 - \|x_{new} - x_{old}\|_1 \geq 0 \;\wedge\; \Delta p \, \|a_{old}\|_1 - \|a_{new} - a_{old}\|_1 \geq 0 \tag{S105}$$

   with typically $\Delta p = 10^{-6}$ and the L^1-norm $\|.\|_1$. If the algorithm had not converged, yet, we would return to Step 2.

Further technical comments: We discretised time with a step size of $\Delta t = 0.1$, so all quantities are defined on that time grid. For steps 2 and 3 we used the default numerical ordinary differential equation (ODE) solver of the integrate.odeint function in the scipy Python package [9] which uses the lsoda solver from the Fortran library odepack. The algorithm uses an adaptive step size but has dense output, which means that it outputs its solution with the same fixed time step, $\Delta t = 0.1$ in our case, regardless of the time steps used in the integration algorithm. The ODE solver expects to be able to evaluate the right hand side of the corresponding ODE at arbitrary times. Since we work with discretised quantities, we wrapped them in linear interpolating functions before passing them into the ODE. Furthermore, we instructed the algorithm to not use integration time steps larger than 0.5. In order to avoid stability issues with spurious sign changes when i became very small, we performed a variable transform and solved the ode for $\ln i$ instead, which is guaranteed to always correspond to positive i.

When calculating the Nash equilibrium, the forward-backward sweep can be used as is. The government intervention $\varepsilon(t)$, if present, enters as an explicit time dependence

into the running cost C. The control is κ for which we used either $\kappa = \kappa^*$, $\kappa = 1$, or the final converged κ from a previous calculation with similar parameters as the initial guess. When calculating the optimal government strategy, the forward-backward sweep is used in nested form. In the outer sweep, the government intervention field ε represents the control, for which we used either $\varepsilon = 0$ or the final converged ε from a previous calculation with similar parameters as the initial guess. At each iteration of the government intervention, we run the inner sweep for the Nash equilibrium with κ as the control until convergence, with the current iteration of the government intervention acting exogenously. When the outer sweep converges, it self-consistently yields the optimal government intervention and the corresponding Nash equilibrium population behaviour.

D Utility salvage term for vaccination at t_f

Assume that a perfect vaccine becomes available at t_f, which immediately protects every susceptible individual, $s(t > t_f) = 0$ and $\psi_s(t > t_f) = 0$. The SIR dynamics of eq. (1) in the main text then reduce to

$$\dot{i} = -i \tag{S106}$$

Therefore, the population of infectious $i(t_f) = i_f$ recover as

$$i(t > t_f) = i_f \exp[-(t - t_f)] \tag{S107}$$

and likewise for the individual dynamics

$$\psi_i(t > t_f) = \psi_{i,f} \exp[-(t - t_f)] \tag{S108}$$

Since there is no danger of becoming newly infected after t_f, there is no reason to modify one's behaviour, $\kappa(t) = \kappa^*$. Taking the utility defined by eq. (5)

$$U = \int_0^\infty u(t)dt \text{ with}$$
$$u = f^{-t}\left[-\alpha\,\psi_i(t) - (\kappa(t) - \kappa^\star)^2 + \varepsilon(t)(\kappa(t) - \kappa^\star)\right]$$

we identify the late time utility coming from $t > t_f$ as

$$U_f = -\int_{t_f}^\infty f^{-t}\alpha(i)\psi_i\,\mathrm{d}t \tag{S109}$$

For $t > t_f$, we assume $\alpha(i) = \alpha(0)$ constant (strictly speaking, in the case where we apply a tanh-form for $\alpha(i)$, this is only an approximation but is accurate to leading order in $i_f \ll i_{hc} \ll 1$). This can immediately be integrated to yield

$$U_f = -f^{-t_f}\alpha(0)\,\frac{\psi_{i,f}}{1 + \log f} \tag{S110}$$

The boundary conditions are therefore

$$v_s(t_f) = \frac{\partial U_f}{\psi_{s,f}} = 0$$
$$v_i(t_f) = \frac{\partial U_f}{\psi_{i,f}} = -f^{-t_f}\alpha(0)\,\frac{1}{1 + \log f} \tag{S111}$$

Assuming Nash equilibrium, the salvage term reads

$$U_f = -f^{-t_f}\alpha(0)\,\frac{i_f}{1 + \log f} \tag{S112}$$

References

1. Gelfand IM, Fomin SV. Calculus of Variations. Dover Publications; 2000.

2. Sussman GJ, Wisdom J. Structure and Interpretation of Classical Mechanics. MIT Press, Cambridge; 2014. Available from: https://tgvaughan.github.io/ sicm/.

3. Arnold VI. Mathematical Methods of Classical Mechanics. vol. 60 of Graduate Texts in Mathematics. 2nd ed. Axler S, W GF, Ribet KA, editors. Springer; 1989.

4. Bechhoefer J. Control Theory for Physicists. Cambridge University Press; 2021.

5. Chachuat BC. Nonlinear and Dynamic Optimization: From Theory to Practice. Automatic Control Laboratory, EPFL, Switzerland; 2007.

6. Lenhart S, Workman J. Optimal Control Applied to Biological Models. Chapman and Hall/CRC; 2007.

7. Pontryagin LS, Boltyanskii VG, Gamkrelidze RV, Mishchenko EF. The Mathematical Theory of Optimal Processes. Gordon and Breach Science Publishers; 1986.

8. Nocedal J, Wright SJ. Numerical Optimization. 2nd ed. Springer; 2006.

9. Virtanen P, Gommers R, Oliphant TE, Haberland M, Reddy T, Cournapeau D, et al. SciPy 1.0: fundamental algorithms for scientific computing in Python. Nature Methods. 2020;17(3):261–272. doi:10.1038/s41592-019-0686-2.
